# Hepatocellular Carcinoma: Current Management and Future Development—Improved Outcomes with Surgical Resection

**DOI:** 10.4061/2011/728103

**Published:** 2011-06-23

**Authors:** Yoji Kishi, Kiyoshi Hasegawa, Yasuhiko Sugawara, Norihiro Kokudo

**Affiliations:** Division of Surgery, Depatments of Hepatobiliary Pancreatic Surgery and Artificial Organ and Transplantation, Graduate School of Medicine, The University of Tokyo, 7-3-1, Hongo, Bunkyo-ku, Tokyo 113-8655, Japan

## Abstract

Currently, surgical resection is the treatment strategy offering the best long-term outcomes in patients with hepatocellular carcinoma (HCC). Especially for advanced HCC, surgical resection is the only strategy that is potentially curative, and the indications for surgical resection have expanded concomitantly with the technical advances in hepatectomy. A major problem is the high recurrence rate even after curative resection, especially in the remnant liver. Although repeat hepatectomy may prolong survival, the suitability may be limited due to multiple tumor recurrence or background liver cirrhosis. Multimodality approaches combining other local ablation or systemic therapy may help improve the prognosis. On the other hand, minimally invasive, or laparoscopic, hepatectomy has become popular over the last decade. Although the short-term safety and feasibility has been established, the long-term outcomes have not yet been adequately evaluated. Liver transplantation for HCC is also a possible option. Given the current situation of donor shortage, however, other local treatments should be considered as the first choice as long as liver function is maintained. Non-transplant treatment as a bridge to transplantation also helps in decreasing the risk of tumor progression or death during the waiting period. The optimal timing for transplantation after HCC recurrence remains to be investigated.

## 1. Introduction

Surgical resection is currently the standard option and treatment of first choice for hepatocellular carcinoma (HCC), given appropriate patient selection. The safety of surgical resection has been established over the last few decades, and the mortality rate after hepatic resection in experienced centers is less than 5%. The indications for surgical resection have expanded even to advanced HCCs, as complicated hepatic resection can be safely performed owing to advances in the surgical techniques. At the same time, the minimally invasive approach, namely, laparoscopic hepatectomy, has also come to be increasingly advocated over the last decade. The safety and feasibility of laparoscopic hepatectomy after the learning curve period has been established in high-volume institutions, and the indications have been expanded from partial resection of benign tumors to major resection of malignant tumors, including HCCs. In this paper, the roles of aggressive hepatic resection for advanced HCC, the role of the recently advocated minimally invasive hepatectomy, the role of preoperative and postoperative adjuvant treatments, and the importance of a multimodality approach, including local ablation therapy, transarterial chemoembolization (TACE), and liver transplantation, are described. 

## 2. Expansion of the Indications for Surgical Resection

Curative surgical resection is currently the only strategy for achieving a potentially satisfactory long-term outcome in patients with HCC and should, therefore, be the treatment of first choice as long as the tumor is judged to be resectable. Here, the term “resectability” is subjective and its definition varies according to the surgeons' skill. Several criteria to decide the indication for surgical resection based on the liver function and tumor status have been proposed. The method adopted for preoperative evaluation of liver function, which would determine the extent of resection of the liver, may vary among districts and institutions. In most Western countries, the presence/absence of portal hypertension is emphasized as an important criterion, which is estimated along with the Child-Pugh class, and is diagnosed based on the findings in hepatic venous pressure gradient, radiological images of splenomegaly and abdominal collaterals, thrombocytopenia (platelets <100,000/mm^3^), and presence of esophagogastric varices [1, 2]. One of the classical staging systems under the Barcelona Clinic Liver Cancer (BCLC) criteria included these criteria for the selection of appropriate treatments and recommended hepatic resection only for patients with a solitary HCC without portal hypertension [3]. Furthermore, this criterion is also included in the guideline for the treatment of HCC established by the American Association for the Study of Liver Diseases and European Association for the Study of the Liver [2, 4]. In contrast, several studies reported the experiences of resection of multinodular HCCs or HCCs with portal hypertension yielding survival benefits, especially in patients where the background liver cirrhosis was classified as Child-Pugh class A [5, 6].

On the other hand, in Asian countries indocyanine green retention at 15 minutes (ICG-R15) is used for patient selection [3, 7, 8]. ICG-R15 is included in the preoperative evaluation of liver damage, along with determination of the presence/absence of ascites, serum bilirubin, serum albumin, and prothrombin time [9]. Recently published Japanese evidence-based clinical guidelines for the diagnosis and treatment of HCC propose surgical resection as the treatment of first choice for HCC patients with 3 or fewer nodules of any size categorized into liver damage of A or B [10]. This guideline recommend either surgical resection or local ablation therapy for early HCC [10]; however, it still remains under debate as to which of the two modalities might be more appropriate. Two randomized controlled trials (RCTs) comparing surgical resection and ablation therapy for small HCCs have been reported, and both yielded similar therapeutic results [11, 12]. However, there were critical flaws in both studies, including an insufficient number of patients, imbalance in the background characteristics, and high conversion rates; therefore, the results of these two RCTs are invalid [13]. In 2010, Huang et al. reported the results of an RCT of hepatectomy and radiofrequency ablation (RFA) for patients with HCC fulfilling the Milan criteria (single tumor ≤5 cm, or two or three tumors with none >3 cm) [14] and categorized as Child-Pugh A or B. Each of the hepatectomy and RFA groups included 115 patients. Although the tumor size was larger in patients in the hepatectomy group, the prognosis was significantly better in this group than that in the RFA group (5-year survival rate: 76% versus 55%). Further analyses by subgroup in patients with solitary HCC measuring 3 cm or less, solitary HCC measuring 3 to 5 cm, multinodular HCCs, or HCC patients with severe liver cirrhosis showed significantly better survival in the patients undergoing hepatectomy in all subgroups [15]. 

For more advanced HCCs, such as huge tumors measuring more than 10 cm in diameter, tumors associated with macroscopic vascular invasion, or tumors with extrahepatic metastases, surgical resection is advocated, because no other treatments such as local ablation, systemic chemotherapy, or liver transplantation are effective. Portal vein embolization (PVE), which was originally proposed as a strategy to decrease the risk of hepatic failure after extended hepatectomy for hilar cholangiocarcinoma [16], also has a role in increasing the patient suitability for hepatic resection in patients with HCC [17]. Although the regenerative capacity of the cirrhotic liver may be poor, the combination of PVE with TACE [18, 19], further combined with hepatic vein embolization, may help in increasing the patient suitability for hepatectomy [20]. Although studies encouraging aggressive surgery for advanced HCCs, as described below, were all retrospective in nature and showed good results in selected patients, surgery should be advocated if the safety is guaranteed by a low mortality and morbidity, because no other treatment has been demonstrated to be potentially as effective. 

### 2.1. Large HCCs

Several studies have reported the short- and long-term outcomes of resection of extremely large (>10 cm in diameter) HCCs [21–24]. In these studies, 40 to 166 patients with HCC tumors measuring 10 cm or more in diameter were evaluated. Although relatively extensive hepatic resection was required, associated with an increased blood loss, the postoperative morbidity and mortality were comparable with those of hepatectomy for smaller HCCs. The mortality and 5-year overall survival rates were 2–3.3% and 28–33%, respectively. Multiple tumors, vascular invasion, and impaired liver function were found to be predictors of poor survival. The safety and survival benefits shown in selected patients in these studies may justify aggressive resection for large HCCs.

Large HCCs should be treated by surgical resection as long as the liver function is maintained within a satisfactory range. Recently, nonalcoholic fatty liver disease (NAFLD) has been increasingly identified as a cause of HCC. Paradis et al. [25] compared the pathologic features of HCCs associated with metabolic syndrome or arising in a background of cryptogenic cirrhosis with those arising in a background of chronic liver disease. The former are larger in size, more frequently well differentiated and the background liver is less fibrotic. Therefore, the opportunity to resect indications for resection in patients with large HCCs is expected to increase. 

### 2.2. HCCs with Macroscopic Vascular Invasion

Presence/absence of macroscopic vascular invasion is one of the strongest predictors of the prognosis in patients with HCC. HCCs tend to invade adjacent venous tributaries, which is associated with an increased risk of intrahepatic or extrahepatic metastases [26–28]. Resection of HCCs with macroscopic vascular invasion or tumor thrombi is technically challenging and has been considered to yield limited survival benefit. However, the prognosis of HCCs with vascular invasion is even more dismal if they are left untreated. The median survival of untreated HCC patients with portal venous invasion is only 2.7 months [29]. Surgical resection leads to better survival outcomes than nonsurgical treatment strategies [30]. Several studies have shown poor survival benefit of surgical resection in HCC patients with portal venous invasion of the main trunk (Vp4 [9]) or of a first-order branch (right or left main trunk, Vp3) as compared with that in HCC patients with portal venous invasion of the secondary tributaries (Vp2) or further peripheral tributaries (Vp1) of the portal vein, and resection for HCCs with Vp3 or Vp4 is not recommended [31, 32]. On the other hand, some authors have advocated aggressive resection even for HCCs with Vp3 or Vp4, reporting 5-year survival rates ranging from 10.9% to 42% [33–38]. Minagawa et al. previously reported the outcomes of 18 selected HCC patients with Vp3 or Vp4 treated by preoperative TACE followed by anatomical hepatic resection and reported a 5-year survival rate of 42% [34]. Based on the results of multivariate analyses, they proposed selection criteria for TACE followed by resection as no more than two nodules, a nonoccluded portal vein trunk, and an ICG-R15 of less than 20%. As for the techniques for resection of a portal vein tumor thrombus, two types of procedures have been reported. One is resection of the involved segment of the portal vein; Wu et al. insisted on the surgical margin being secured by this technique [33]. The other is the peeling-off technique, in which a portal venotomy is placed and the tumor thrombus is detached and removed from the internal wall of the portal vein. Inoue et al. showed comparable postoperative overall and recurrence-free survivals between this peeling-off technique and the en bloc portal vein resection (5-year overall survival: 39% versus 41%, *P* = .90; 5-year recurrence-free survival: 23% versus 18%, *P* = .89) [37].

As compared to portal venous invasion, HCC invasion of the bile duct or inferior vena cava is quite rare. In regard to the prognostic influence of bile duct tumor thrombi, Satoh et al. previously reported that there was no significant difference in the postoperative prognosis between patients with and without bile duct thrombi [39]. However, this result seemed to be influenced by the small number of patients with bile duct thrombi. More recently, Noda et al. reported the results of surgical resection of 22 patients with biliary tumor thrombi; they reported a 3-year survival rate of 30%, which was significantly worse than that of patients without biliary tumor thrombi. In the present study, portal or hepatic vein tumor thrombi, seen in 13 patients (59%), was the only significant predictor of the prognosis [40]. Ikenaga et al. showed that bile duct invasion was associated with a worse prognosis irrespective of the degree of invasion, that is, patients with biliary invasion only to a third-order or more peripheral branch showed a similarly poor prognosis to those with more proximal bile duct invasion (first- or second-order branches or the common hepatic duct) [41]. Most of these studies advocated aggressive hepatic resection in selected HCC patients with macroscopic vasculobiliary invasion; however, additional preoperative or adjuvant treatments should be considered to improve the long-term outcomes. 

### 2.3. Resection of Extrahepatic Metastases

Poon et al. reported limited benefit of aggressive resection of extrahepatic metastases when the extrahepatic lesion was solitary and/or the intrahepatic recurrence was well controlled [42]. Yang et al. reported that extrahepatic recurrence occurring after repetitive treatment for intrahepatic recurrence was associated with a better prognosis than simultaneous intrahepatic and extrahepatic recurrence or extrahepatic recurrence preceding intrahepatic recurrence; however, the 5-year survival rate after initial hepatic resection in 20 patients with the former type of extrahepatic recurrence was only 30% [43].

The lungs are the most common site of extrahepatic metastasis from HCC, with pulmonary metastases accounting for 50–60% of all extrahepatic metastases [44, 45]. The outcomes of surgical resection of lung metastases have been increasingly reported recently, with the reported 5-year survival rate after pulmonary resection being in the range of 27–33%; however, the number of patients included in each of these studies was small (8–61 patients), because, in most cases of pulmonary metastases, the lesions are multiple and surgical resection is contraindicated [46–49]. Kawamura et al. suggested that surgical resection may be considered in patients with up to three pulmonary lesions [49]. Kuo et al. showed that the disease-free interval and number of lesions were associated with the prognosis [47].

Surgical resection of other sites than the lung is reported only sporadically. Recently, Chan et al. reported the outcomes of surgical resection of extrahepatic metastases including in the lung, bone, brain, soft tissues, and heart and showed that resection of lung metastases was associated with the most favorable prognosis. Although the outcomes of resection of bone or brain metastases were dismal, with a 3-year survival rate of 9% and 0%, respectively, even in these groups, patients who underwent surgical resection showed better survivals than patients who did not undergo surgical resection [50]. Sakamoto et al. reported four cases of resection of adrenal metastases. A literature review of 79 cases of adrenal metastases did not reveal any significant survival benefit of surgical resection. However, considering the risk of tumor rupture and venous invasion resulting in pulmonary embolism, or the technical difficulty of TACE due to the presence of three arteries feeding the adrenal grand, adrenalectomy may be a valid strategy, because it can be performed safely with minimal mortality [51]. Until other treatment alternatives are established to improve the patients' quality of life and life expectancy, surgical resection may be justified for the treatment of extrahepatic metastases. 

## 3. Anatomic Resection

As described above, HCCs show a high predilection for invading the adjacent portal vein and, consequently, intrahepatic metastasis. On the other hand, extensive hepatic resection is contraindicated in most cases due to the severity of the background liver disease. To overcome this problem, Makuuchi et al. proposed anatomic subsegmentectomy [52]. In this type of hepatectomy, intraoperative ultrasonography is performed to identify the locations of the hepatic tumors. Dye (indigo carmine) is injected into the portal venous tributaries supplying the region containing the tumor. The area showing positive staining is then marked with electrocautery, and a parenchymal transection is performed. According to the location or size of the tumor and the background liver function, a part of one Couinaud's segment or more than one segment is resected. The influence of anatomic resection on the postoperative prognosis has been evaluated recently. Most studies have shown the superiority of anatomic resection for prolonged overall or recurrence-free survival, especially in selected patients with solitary HCCs, small HCCs, or HCCs fulfilling the Milan criteria [14, 53–57]. Among the studies, the largest series was a Japanese nationwide survey including 5781 patients who underwent anatomic subsegmentectomy or nonanatomic minor hepatectomy for solitary HCCs. The overall survival was marginally better in the anatomic resection group in the overall analysis (*P* = .053). Subgroup analysis showed significantly better disease-free survival following anatomic resection in patients with HCCs measuring 2 to 5 cm in diameter (*P* = .0005) [56]. 

## 4. Resection of Recurrent HCCs

The most common site of recurrence is the remnant liver, which accounts for 85% to 90% of the initial recurrences [58]. The reported incidence of intrahepatic recurrence within 2 years after primary hepatic resection is 70% [58]. The effectiveness of systemic chemotherapy for HCCs has not yet been established; therefore, in the absence of extrahepatic metastases, local treatment is currently the only approach that can yield long-term survival in patients with recurrent HCC. Several studies have demonstrated that repeat hepatectomy yields a better prognosis than other nonsurgical treatments, with a 5-year survival rate after repeat hepatectomy of around 50–70%, which is almost comparable to the survival rate after first hepatectomy [59–63]. Wu et al. reviewed the outcomes of patients who underwent hepatectomy for HCC up to four times and showed that patients undergoing their second and third hepatectomies, but not those undergoing the fourth hepatectomy, showed significantly better survivals than those who did not undergo repeat hepatectomy [64]. All of these studies, however, were retrospective in nature, and the selection bias for patients who underwent repeat hepatic resection must be considered. Repeat hepatic resection is indicated for only a limited proportion of patients, because multiple intrahepatic tumor recurrences and/or impaired liver function due mostly to the background liver diseases represent contraindications to repeat hepatectomy. Minagawa et al. analyzed the prognostic predictors in patients undergoing repeat hepatectomy and reported a solitary HCC at primary hepatectomy, disease-free interval of 1 year or more, and absence of portal venous invasion at the second hepatectomy as independent predictors of a favorable prognosis [65]. Repeating locoregional treatment such as ethanol injection (PEI), radiofrequency ablation (RFA), or TACE, for an intrahepatic recurrence may also help in prolonging patient survival [66–70]. Locoregional treatments may be repeated as long as there are no extrahepatic recurrences and the remnant liver function is reasonably adequate. Surgical resection after PEI/RFA can also be considered for recurrent HCC [71]. 

## 5. Preoperative and Adjuvant Treatments

Unlike the case for colorectal liver metastases, in which preoperative chemotherapy may improve the prognosis of patients with either resectable or initially unresectable tumors [72–75], there is no established preoperative adjuvant strategy to improve the prognosis in HCC patients. Previous studies have evaluated the role of preoperative TACE, but the long-term outcomes remain controversial. Most studies, including one RCT, have shown no survival benefit [76–82]. Furthermore, some studies identified preoperative TACE as a predictor of extrahepatic metastasis [83, 84] or worse survival [79, 85]. On the other hand, selected patients with TACE-mediated complete necrosis showed a significantly more favorable prognosis after hepatic resection [77, 83, 85–88]. These results may be explained by Adachi's hypothesis that viable cancer cells are less firmly attached and are more likely to disseminate into the blood stream during surgical manipulation following incomplete necrosis by TACE [86]. Considering the low incidence of complete necrosis achieved by TACE, ranging from 6% to 29% according to previous reports [77, 87, 88], routine implementation of preoperative TACE may not be recommended. Several other studies report that preoperative TACE may improve the disease-free and overall survivals in selected cases, such as HCC patients with macroscopic portal venous invasion [34], advanced-stage tumors [89], severe liver dysfunction (ICG-R15 ≧ 17%) [90], or centrally located large tumors [84]. One study showed that TACE converted initially unresectable HCCs to resectable tumors, yielding a 5-year survival rate of 56% [91]. Taking all the above reports into consideration, TACE as a postoperative adjuvant treatment seems to have a role in improving the prognosis of patients, especially of those having advanced HCCs with portal venous invasion or intrahepatic metastases [92, 93].

A multitargeted agent sorafenib, which exerts both antiangiogenic and antiproliferative effects, is the first systemic agent to have yielded survival benefit in patients with advanced HCCs in a phase III, randomized, placebo-controlled trial [94]. However, the benefit is limited (median overall survival: 10.7 months versus 7.9 months) and treatment with this drug has not yet been validated as a preoperative or adjuvant treatment. Until date, other modalities such as hepatic artery infusion of radionuclide Yttrium-90 microspheres, or combined subcutaneous interferon alpha and intraarterial infusion chemotherapy, seem to be more promising strategies than sorafenib treatment to downstage advanced HCCs, including cases with macroscopic portal venous invasion [95–100]. Especially, two recent reports from Japanese groups showed a response rate of 33–52% following combined interferon and intra-arterial 5-fluorouracil therapy in advanced HCC patients with portal venous invasion. These results suggest further increase in the number of candidates suitable for surgical resection among patients with initially unresectable HCCs. 

## 6. Laparoscopic Hepatectomy

Laparoscopic liver resection was first reported in the early 1990s for partial resection of segment 6 for a 6 cm focal nodular hyperplasia and wedge resection of segment 5 for colorectal liver metastases [101]. Since then, the number of reported cases of laparoscopic liver resection has increased dramatically, especially over the last 5 years [102]. Although some surgeons are still skeptical about the oncological curative potential of laparoscopic surgery and evaluation of the long-term prognosis is required to justify the minimally invasive approaches for hepatic malignancies, several specialized centers have expanded the indications of laparoscopic hepatectomy from benign tumors to malignant tumors, including HCCs [103–105] and from wedge resection of the anterolateral segments to major hepatectomy or resection of the posterosuperior segments, such as segment 8, 7, or 1 [106, 107]. The consensus conference by 45 experts held in October 2008 proposed that the most suitable candidates among HCC patients for laparoscopic hepatectomy are, in general, those with solitary lesions measuring 5 cm or less in diameter, located in the peripheral segments. The conference also proposed that major hepatectomy or other technically complicated procedures should be left to experienced surgeons [108].

Several surgeons advocate laparoscopic resection for HCC, especially that in a cirrhotic liver, due to its less-invasive characteristic, because less liver mobilization is required and the amount of intravenous fluid needed is reduced due to the minimized insensible fluid loss occurring during this operation as compared with that during open liver resection. Fluid accumulation in the third space decreases, which may be expected to be associated with a reduced risk of prolonged postoperative accumulation of ascites [109–112]. On the other hand, higher morbidity and mortality have been reported, even from highly specialized institutions [113]. As for the survival outcomes, only a few reports based on studies of a small number of cases each have been published, which have shown a comparable short-term prognosis in patients undergoing laparoscopic hepatectomy as compared to that in patients undergoing open hepatectomy [109, 111, 112]; further investigation is required for validation. A significant learning curve is inevitable to establish the safety and feasibility of laparoscopic surgery [114] and this is one of the major limitations in relation to the popularization of laparoscopic hepatectomy. The indications for laparoscopic resection should be appropriately decided according to the types and conditions of the tumors and the technical skills of the surgeons. More importantly, the safety and oncological curative potential must be accorded priority; therefore, conversion to an open procedure should be expedited if bleeding cannot be controlled laparoscopically, or an adequate resection margin cannot be obtained, or adhesions preclude the laparoscopic procedure [102, 113]. 

## 7. Liver Transplantation for HCCs

The Milan criteria, which were published in 1996 [14], have served as appropriate selection criteria for patients with HCC who are potential candidates for liver transplantation. The criteria have been validated by numerous subsequent studies, with patients fulfilling the Milan criteria showing significantly better survivals than those not fulfilling the criteria, in both cadaveric and living donor liver transplantation (3-year survival rate of 79–91% versus 60–66%) [115, 116]. The criteria are also utilized by the United Network for Organ Sharing [117]. However, the Milan criteria are rather restrictive, permitting liver transplantation in only a limited proportion of patients with HCC, and therefore, expansion of the criteria has been proposed. The most representative example of such expanded criteria is the University of San Francisco (UCSF) criteria proposed by Yao et al., which include solitary tumor ≦ 6.5 cm, or two or three nodules with the largest lesion ≦ 4.5 cm and total tumor diameter ≦ 8 cm [118]. These criteria were also validated by subsequent studies from high-volume transplantation centers, which reported overall 5-year survival rates of 52–64% [119, 120]. In cases of living donor liver transplantation, it may be possible to expand the selection criteria more readily, because the donors would be expected to have a stronger motivation for self-giving and dedication to the recipients. In The University of Tokyo, patients with HCC having up to 5 nodules with a maximal diameter of 5 cm (the 5-5 rule) have been treated by transplantation, and the 5-year survival rate in a series of 72 patients treated according to this 5-5 rule was reported to be 75% [121]. Other Japanese institutions have reported a more aggressive approach to living donor liver transplantation, operating on HCC patients with no extrahepatic metastases or macroscopic vascular invasion, but regardless of the size and number of tumors [122–124]. Soejima et al. have insisted that patients with small multiple HCCs should be included as candidates for LT [122]. In their report of 60 HCC patients who underwent living donor liver transplantation, 23 patients with 4 to 10 tumors and 12 patients with more than 10 tumors showed 3-year recurrence-free survival rates of 75% and 72%, respectively, and only preoperative des-gamma-carboxy prothrombin (DCP) levels of >300 mAU/ml and tumor size >5 cm were identified as independent prognostic factors. They validated this result recently in an extended series of 90 patients. In this study, a 5-year survival rate of 83% was seen in 85 HCC patients meeting the criteria of a maximal tumor size of less than 5 cm or DCP level of less than 300 mAU/ml [124]. Similarly, Ito et al. analyzed the prognostic factors in their series of 125 patients and proposed the following criteria: 10 or fewer tumors, with each tumor measuring 5 cm or less in diameter, and a DCP level of 400 mAU/ml or less. The 5-year survival rate in 78 patients meeting these criteria was 87% [123].

In most Western countries as in Japan, hepatitis C virus (HCV) is the most common etiological agent associated with HCC. Hepatitis C recurs inevitably in the transplanted liver, and progression to graft cirrhosis occurs more rapidly under immunsuppression [125], which becomes another possible cause of the patients' death in addition to tumor recurrence. Therefore, a special postoperative immunosuppression protocol, such as a steroid-free protocol [126, 127], or cyclosporine-based instead of a tacrolimus-based protocol should be considered [128]. Preemptive antiviral therapy using interferon and ribavirin may also help in improving the survival of the recipients, by reducing the risk of progression of fibrosis in the grafts [129, 130].

Under the current circumstance of shortage of donors, opportunities for liver transplantation are limited. Therefore, nontransplant treatments, including open or laparoscopic hepatic resection, PEI/RFA, or TACE, should be the treatment modalities of first choice in HCC patients with compensated liver cirrhosis. Even in patients with decompensated cirrhosis, these modalities may well be used as a bridge to transplantation. Several studies have shown that secondary or salvage transplantation for downstaged HCC by nontransplant treatment or for recurrent HCC after initial treatment results in acceptable survival outcomes, especially if the HCC meets the Milan criteria prior to transplantation [131–134]. The reported 5-year survival rates after secondary transplantation from these studies are in the range of 60–80%. Interestingly, Takada et al. reported that patients with one or two non-transplant treatments prior to liver transplantation showed better outcomes after transplantation than patients without any previous treatment and also patients with 3 or more treatments. They concluded that patients developing recurrent HCCs should be referred for transplantation before further nontransplant treatment is repeated [135]. Further investigation is required to determine the appropriate timing of transplantation. 

## 8. Conclusions

The indications for surgical resection in patients with HCC have expanded concomitantly with the establishment of the safety and feasibility of aggressive resection. However, a high incidence of postoperative recurrence is still an obstacle to the achievement of long-term survival in advanced HCC patients. A multimodality approach is required, especially for patients with recurrent HCC or HCC with background liver cirrhosis, which preclude hepatic resection. The benefit of systemic chemotherapy for extrahepatic metastases is still limited, and the development of better pharmacological agents in the future is expected. Most studies referred to in this paper were retrospective in nature. Although RCTs may not be ethically appropriate in all clinical researches, more prospective studies are needed to accumulate evidence for the establishment of multidisciplinary treatments for HCC. 
